# Chronic Traffic-Induced PM Exposure and Self-Reported Respiratory and Cardiovascular Health in the RHINE Tartu Cohort

**DOI:** 10.3390/ijerph6112740

**Published:** 2009-10-30

**Authors:** Hans Orru, Rain Jõgi, Marko Kaasik, Bertil Forsberg

**Affiliations:** 1 Department of Public Health, University of Tartu, Ravila 19, Tartu, 50411, Estonia; 2 Department of Public Health and Clinical Medicine, Umea University, Umea, SE-901 87, Sweden; E-Mail: Bertil.Forsberg@envmed.umu.se; 3 Lung Clinic, Tartu University Hospital, Riia 167, Tartu, 51014, Estonia; E-Mail: Rain.Jogi@kliinikum.ee; 4 Institute of Physics, University of Tartu, Riia 142, Tartu, 51014, Estonia; E-Mail: Marko.Kaasik@ut.ee

**Keywords:** air pollution, particulate matter, traffic, respiratory diseases, cardiovascular diseases

## Abstract

The relationship between exposure to traffic induced particles, respiratory health and cardiac diseases was studied in the RHINE Tartu cohort. A postal questionnaire with commonly used questions regarding respiratory symptoms, cardiac disease, lifestyle issues such as smoking habits, indoor environment, occupation, early life exposure and sleep disorders was sent to 2,460 adults. The annual concentrations of local traffic induced particles were modelled with an atmospheric dispersion model with traffic flow data, and obtained PM_exhaust_ concentrations in 40 × 40 m grids were linked with home addresses with GIS. The relationship between the level of exhaust particles outside home and self-reported health problems were analyzed using a multiple logistic regression model. We found a significant relation between fine exhaust particles and cardiac disease, OR = 1.64 (95% CI 1.12–2.43) for increase in PM_exhaust_ corresponding to the fifth to the 95th percentile range. The associations also were positive but non-significant for hypertension OR = 1.42 (95% CI 0.94–2.13), shortness of breath OR = 1.27 (95% CI 0.84–1.94) and other respiratory symptoms.

## 1. Introduction

Numerous epidemiological studies have shown an association between particulate air pollution and cardiopulmonary health [1]. Fine exhaust particles are believed to be the most harmful [2–5]. It has been assumed that traffic-induced particles emitted from combustion processes are more potent in posing adverse health effects than those from non-combustion processes. Besides exhaust emissions, the major types of traffic particles are brake wear, tyre wear, road surface abrasion and re-suspension [2]. However, meta-analysis of epidemiological studies differentiating between the coarse particles PM_2.5–10_ and the fine particles PM_2.5_, has shown that health effects exist from both fractions [3].

Traffic air pollution has been associated both with respiratory and cardiovascular effects. The cardiovascular effects of air pollution include myocardial ischemia, atherosclerosis, infarctions, heart failure, arrhythmias, strokes etc., and these are said to be the leading causes of air pollution induced morbidity and mortality [4]. Many of the studies have shown that chronic PM exposure significantly increases cardiovascular but not pulmonary mortality risk [5,6]. However, several other studies have shown that long-term PM exposure is also associated with deficiencies in lung function and increased symptoms of obstructive airway disease, such as chronic cough, bronchitis, chest illness, etc. [1]. It is possible that morbidity is more important in the context of PM exposure and respiratory health.

Some studies have associated the levels of vehicle exhaust outside the home with an increase in the incidence of asthma [7,8]. The prevalence of diagnosed asthma and asthma-like symptoms is low in Estonia, both among children [9] and adults [10], while the prevalence of non-specific respiratory symptoms [10] and bronchial hyper-responsiveness [11] is relatively high. This has raised some scepticism about the validity of doctor diagnosed asthma in Estonia [12]. However, repeated cross-sectional studies in children have shown an increase in asthma prevalence in children [13] while longitudinal analysis of ECRHS cohort has shown low incidence of asthma among adults [14].

Traffic pollution has been linked with other respiratory symptoms as well. It has been associated with the prevalence of chronic bronchitis [15,16], coughing attacks, and wheezing [17] and rhinitis [18]. The links to chest tightness [19] and shortness of breath [20] are weaker or supposed.

The association between air pollution and blood pressure has also been demonstrated in several studies, but these have mostly investigated short term increases of ambient air pollutants [21]. However, some studies have found no association, particularly with long term exposure [22,23]. Nevertheless, vehicle pollution may be an important risk factor. One study reported a stronger association between PM_2.5_ and high blood pressure in the presence of roadway traffic [24], and Künzli *et al.* [24] have found association between ambient PM_2.5_ and atherosclerosis in an area with heavy traffic LA. A recent meta-analysis of three cross-sectional surveys found weakly positive associations between cardiovascular disease and PM_10_ [25].

Research into air pollution and health effects in Eastern Europe and especially in the Baltic States is limited. One reason has been the limited pollution data available, especially from earlier years. A major recent epidemiologic study has been conducted in Lithuania, where a relationship between exposure to urban nitrogen dioxide pollution and the risk of myocardial infarction was determined [26]. Lately a model to calculate traffic pollution concentrations in Tartu has been improved [27]. The aim of our study is to investigate the association between the prevalence of respiratory symptoms, cardiac disease and hypertension and traffic-induced particulate air pollution in the RHINE Tartu cohort in Estonia.

## 2. Material and Methods

### 2.1. Study Population and Site

In 2000/2001 a postal questionnaire was sent to 2,460 adults in Tartu that had answered the ECRHS (European Community Respiratory Health Survey, www.ecrhs.org) questionnaire sent to a random sample of people aged 25–45 in 1993. This follow up in Tartu and six other ECRHS centres established the RHINE cohort (The Respiratory Health in Northern Europe, www.rhine.nu).

Out of the invited 2,460 persons from Tartu we were able to model the PM_exposure_ from traffic at home for 2,216 respondents. Out of these, 1,708 persons (69%) answered the questionnaire. Complete answers regarding respiratory health, cardiac disease, and blood pressure were obtained for 1,684 of the persons for which particle concentrations could be modelled.

The main characteristics of the study population are shown in Table 1. The mean age of the Tartu RHINE cohort in the follow up was 35 years, they had intermediate body mass index (BMI 24.2). The prevalence of cardiac disease, and hypertension and percentage of smokers was relatively high.

Among the rest of self-reported respiratory symptoms, the prevalence in exposed Tartu study population was highest for cough (40.5%); but also high for wheezing (24%), and chest tightness (16.8%). The prevalences for the whole RHINE populations are in more detail given in Jogi *et al.* [10] and Toren *et al.* [14] papers.

Tartu is a city with 100,000 inhabitants, which makes it the second largest city in Estonia, situated in north-eastern Europe. The air pollution levels are not so high in the European context. According to ECRHS II measurements, the annual average PM_2.5_ concentration in Tartu during the study period was 14.8 μg·m^−3^ [28]. According to Orru *et al.*, [29] traffic contributes about 8% of the total PM_2.5_.

As the capacity of the street network remains limited despite growing traffic, congestion occurs at rush hour on several streets. A large number of cars are more than 10 years old, thus the combustion is often not of the highest standard.

### 2.2. Questionnaire on Respiratory Health and Cardiac Diseases

From the RHINE questionnaire we concentrated on possible air pollution health effect outcomes addressed in following questions:

- **Wheezing** “Have you had wheezing or whistling in your chest at any time in the last 12 months?”, and if so, “Have you had this wheezing or whistling when you did not have a cold?”
- **Cough** “Have you been woken by an attack of coughing at any time in the last 12 months?”
- **Chronic bronchitis symptoms** “Do you cough up phlegm in this way almost every day for at least three months every year?”, and if so, “Have you had periods of this kind for at least two years in a row?”
- **Non-allergic rhinitis** “Have you ever experienced nasal symptoms such as nasal congestion, rhinorrhoea (runny nose) and/or sneezing attacks without having a cold?”, and if “Yes”; “No” to question “Do you have any nasal allergies including hay fever?”
- **Chest tightness** “Have you woken up with a feeling of tightness in your chest at any time in the last 12 months?”
- **Breath shortness** “Have you been woken by an attack of shortness of breath at any time in the last 12 months?”
- **Cardiac diseases** “Do you have any cardiac disease?”
- **Hypertension** “Do you have high blood pressure?”

### 2.3. Air Pollution Exposure

The modelled annual mean levels of locally emitted PM_exhaust_ (exhaust particles) and PM_10_ (also road dust, tyre wear etc.) in 2000 outside the home were used as the exposure variables. The AEROPOL model that is based on Gaussian dispersion algorithm with complementary effects (e.g., reflection of Gaussian plume from the ground and capping inversion, dry and wet removal) was applied for estimating the concentrations. Simulation of long-term average PM concentrations was based on averaging the plumes over climatological distribution of thermal stratification (Pasquill–Gifford stability classes [30]), wind direction and speed. The basic concepts of AEROPOL are described by Kaasik and Kimmel [31]—these are rather similar with widely known models, e.g., ADMS [32] or AERMOD [33]. AEROPOL has been applied and validated for urban [34,35] as well as industrial [36] sources. The PM concentrations were modelled in 40 × 40 m grids and geocoded addresses were linked to concentrations using the GIS software Surfer.

The average traffic flows during evening rush hour were available for 487 street links all over the city. As estimations of vehicle emission factors for Estonia are not available, the factors from other countries are used. For exhaust particles, the emission estimation methodology used by the Finnish Meteorological Institute was applied [37]. The emissions per vehicle-kilometre were calculated as was typical for Helsinki. The non-exhaust particulate matter (from road surface, tyres, brakes etc.) was estimated using the average for Stockholm, as calculated by Eneroth *et al.* [38]. According to their data, almost 90% of non-exhaust emissions originated from resuspension of particles from the street surface. It appears that in northern countries these emissions are roughly 10 times greater than the exhaust emissions because studded tyres are used. As the GDP per capita in Estonia nowadays is exceeding 70% of average level of EU and technical norms correspond completely to EU standards, the quality of the car fleet has been rapidly improved during last 10 years. Nowadays there exist no dramatic differences compared to Nordic countries, including the fact that studded tyres are in use through wintertime.

In the analysis, traffic emissions of exhaust and non-exhaust emissions together are considered as PM_10_ since they depend on traffic flows and show a very strong spatial correlation. However, we also present the results obtained using PM_exhaust_ only, since it is often assumed to be the most toxic fraction of particles due to composition or size or both [39–43].

### 2.4. Statistical Analyses

For the analysis of the relationship between PM exposure at the home address and self-reported health problems in the questionnaire, the multiple logistic regression analysis in SPSS was used. All analyses were adjusted for gender, age, body mass index (BMI), smoking history (current, ex-smoker, never) and, in the case of cardiac diseases, also for self-reported high blood pressure (yes/no).

## 3. Results

### 3.1. Exposure

**Figure 1.**
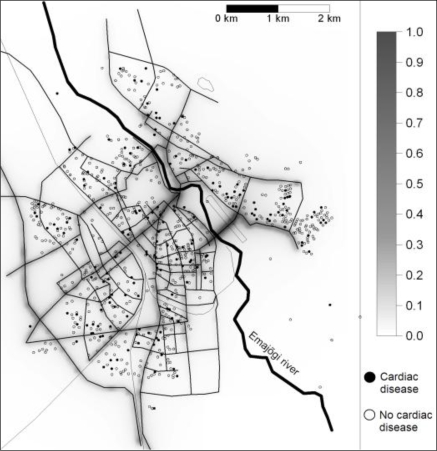
Annual traffic induced PM_exhaust_ exposure (μg·m^−3^) and prevalence of cardiac diseases in 2000 in Tartu.

The mean exposure to local traffic induced exhaust particles (PM_exhaust_) was 0.10 μg·m^−3^ and to any kind of local traffic induced particles (PM_10_) was 0.76 μg·m^−3^. Most of the streets in Tartu have low traffic flows, but some of the streets are quite busy, with elevated vehicle induced pollution levels (max of PM_exhaust_ 0.83 μg·m^−3^ and PM_10_ 7.40 μg·m^−3^) (Figure 1). As the correlation between the annual mean level of PM_exhaust_, and traffic related PM_10_ for included subjects was 0.99 and PM_exhaust_ is often assumed to be the most toxic, relative risks are reported only for PM_exhaust_.

### 3.2. Associations with PM at Home and Prevalences

We found a strong correlation between concentrations of traffic-induced PM and the prevalence odds ratio (OR) for having cardiac disease, and the association became even stronger when we adjusted it for blood pressure (Table 2). For an increase in mass concentration of 1 μg/m^3^ the effect is as high as high OR = 9.04, 95% CI = 1.62–50.42. If the OR for having a cardiac disease is calculated for an increase corresponding to the fifth to the 95^th^ percentile range, it becomes 1.64 (95% CI = 1.12–2.43) for PM_exhaust_.

We did not see any significant association with respiratory symptoms (Table 2). After cardiac disease the OR was highest for hypertension and shortness of breath, problems also related to cardiovascular disease, but these associations were not significant. For people with more risky behaviour such as smokers and ex-smokers and respondents with high BMI (>25), the ORs associated with traffic PM were higher for shortness of breath, lower for cardiac diseases, and approximately the same level as the average for other outcomes.

## 4. Discussion

Since the annual mean levels of vehicle exhaust components and road dust show a high spatial correlation due to a common source, it is not possible to study their unique influence on health within one city. Thus we see our study as an investigation of how traffic related pollution may influence health. We had much better data to model exposure levels in 2000 than for the 1993 ECRHS survey, which is why prevalence at follow up was chosen as the outcome. However, a planned future follow up within the next few years will make it possible to study incidence in relation to pollution levels from 2000 and later.

While the specificity and sensitivity of self-reported asthma and asthma symptoms have been studied a lot [44], we know less about validity of the questions on cardiac diseases and hypertension that were used in the RHINE study. The question used on cardiac diseases has not been validated against medical records, but other similar self-reported conditions have in different ways, at least indirectly. For example “nonspecific” chest pain has been associated with high long-term mortality in a Swedish study [45], and men who recalled a doctor diagnosis of ischemic heart disease, were at extremely high risk of a new major ischemic heart disease event during a follow-up period [46]. Moreover, there are a number of studies where myocardial infarction, angina etc have been studied using questions [47–50] that are rather similar to our questions.

It is somewhat difficult to compare our findings regarding cardiac diseases and traffic-induced particles with other studies, since more specific endpoints like myocardial infarction, arrhythmias, atherosclerosis and ischemic events usually are studied, and the true effect may be different. There are large differences in exposure assessment as well. Rosenlund *et al.* [51] reported ORs in Stockholm County for myocardial infarction and traffic pollution of 1.04 (95% CI = 1.00–1.09) for all cases and 1.16 (95% CI = 1.09–1.24) for fatal cases for an increase in PM_10_ exposure corresponding to the fifth to the 95th percentile. These are slightly lower when compared to our ORs for prevalent cardiac disease, but our Tartu estimates are for a very specific source of pollution. The same reason, and also the fact that the OR was calculated per intertertile change in NO_2_ exposure, could explain the lower ORs in a Lithuanian study [26]. On the other hand, Hoffmann *et al.* [52] indicated that people living within 50 m of major roads have an OR for coronary atherosclerosis through calcification as high as 1.63 (95% CI = 1.14–2.33).

When we adjusted our model for cardiac diseases to also include high blood pressure, the OR increased around 10% (Table 2). However, the positive association between air pollution and high blood pressure was not significant. The reason for this might be that high pressure is usually found to be related mainly to short-term increases in exposure [21]. There are probably other trails than hypertension through what cardiac diseases are developed in the presence of traffic-induced air pollution. Inflammation through pro-oxidative, and pro-inflammatory mediators, interaction of nano-scale particles with cardiovascular systems and autonomic mechanisms in the nervous system are believed to be the main pathways [4]. Moreover, the correlation between hypertension and cardiac diseases is low in our data.

We believe there might be confounders, such as noise and stress, for which the effects are difficult to separate from the effects of air pollution. Noise, including traffic noise, has been associated with stress, sleep disturbance, blood pressure and cardiac diseases [53]. Noise may confound or modify the relation between traffic pollution and cardiac diseases as both are linked to cardiovascular effects [54]. Recent finding have found moderate correlations between traffic-generated air pollution and noise [55], but the published data are limited and somewhat inconsistent. As we had no data regarding traffic noise, we could not adjust for it. Possible confounders also are socioeconomic status, and physical activity for which we had no data. As we have adjusted for BMI and smoking history, these parameters could be indirectly included in the adjustment.

We did not determine any significant associations with respiratory symptoms. However several studies have found such [15–20]. There might be several reasons for our null findings; selection (or survivor) bias could be one. Most people are probably aware that air pollution has a negative effect on asthma and other respiratory diseases. This may cause a selection bias in an epidemiological study—people with respiratory problems may tend to move from polluted streets to less trafficked areas, when more healthy do not. For cardiac diseases the association with air pollution has been observed more recently by researchers and is less known to the general public. The risk of having a selection of people with cardiac diseases away from polluted areas is smaller.

The socioeconomic status may also have influenced the results. There tends to be more cardiac diseases in regions with big block houses compared to regions with residential or small apartment houses. However, the two last groups are very small and no significant difference in regression analysis appeared. These people could have been more stressed etc, what is also important factors inducing cardiac diseases.

## 5. Conclusions

In this study in Tartu a strong relation between exhaust traffic particles and cardiac diseases was established. Per 1 μg·m^−3^ increase in exposure, the exhaust particles due to lower concentrations give much higher ORs compared to PM_10_ from traffic. Per change corresponding to the fifth to the 95th percentile range in exposure, there was a very small difference. We could not see any significant relation to respiratory symptoms or hypertension.

It seems that for local traffic pollution the associations here are much stronger with cardiovascular disease compared to respiratory outcomes. Even if vehicle exhaust particles are a proposed candidate to have more toxic effects, we cannot exclude other components in PM due to the high correlations. Traffic pollution is correlated with noise, which could be an important confounder.

## Figures and Tables

**Table 1.** Characteristics of study population and selected prevalences.

**Age (years)**		**Men**	**Smokers**	**BMI**		**Prevalence (%)**		
**Mean**	**Min-max**	**(%)**		**Mean**	**Min-max**	**Cardiac disease**	**Hypertension**	**Chronic bronchitis**
35	25–50	48	35.3	24.2	15.4–54.9	12.4	12.4	5.7

**Table 2.** Relation between respiratory, and cardiac symptoms and PM_exhaust_ exposure.

	OR^3^ (95% CI^4^)	
	**Per 1 μg·m^−3^ increase in exposure**	**Per increase in exposure corresponding to the fifth to the 95^th^percentile**
Cough^1^	1.01 (0.28–3.64)	1.00 (0.75–1.34)
Chronic bronchitis^1^	0.78 (0.53–11.44)	0.95 (0.87–1.73)
Non–allergic rhinitis^1^	1.79 (0.45–7.17)	1.14 (0.83–1.56)
Wheezing^1^	1.99 (0.36–11.83)	1.17 (0.79–1.75)
Chest tightness^1^	2.34 (0.48–11.48)	1.21 (0.85–1.74)
Shortness of breath^1^	2.92 (0.46–18.65)	1.27 (0.84–1.94)
Cardiac diseases^1^	**7.56 (1.40–40.68)**	**1.58 (1.08–2.31)**
Cardiac diseases^2^	**9.04 (1.62–50.42)**	**1.64 (1.12–2.43)**
Hypertension^1^	4.65 (0.76–28.41)	1.42 (0.94–2.13)

1 Logistic regression model was adjusted for gender, age, body mass index (BMI), and smoking.

2 Logistic regression model was also adjusted for high blood pressure.

3 Odds ratio (OR)

4 Confidence interval (CI)
